# Effects of metabolic parameters’ variability on cardiovascular outcomes in diabetic patients

**DOI:** 10.1186/s12933-023-01848-x

**Published:** 2023-05-15

**Authors:** Subin Lim, Se Hwa Chung, Ju Hyeon Kim, Yong Hyun Kim, Eung Ju Kim, Hyung Joon Joo

**Affiliations:** 1 Division of Cardiology, Department of Internal Medicine, Korea University Anam Hospital, Seoul, Korea; 2 Department of Biostatistics, Korea University College of Medicine, Seoul, Korea; 3 Division of Cardiology, Department of Internal Medicine, Korea University Ansan Hospital, Ansan, Korea; 4 Division of Cardiology, Department of Internal Medicine, Korea University Guro Hospital, Seoul, Korea; 5 Department of Cardiology, Cardiovascular Center, Korea University Anam Hospital, University College of Medicine, 73, Goryeodae-ro, Seongbuk-gu, Seoul, 02841 Korea; 6 Korea University Research Institute for Medical Bigdata Science, College of Medicine, Korea University, Seoul, Korea

**Keywords:** Metabolic variability, Diabetes mellitus, Blood pressure, Lipid profile

## Abstract

**Background:**

Metabolic abnormalities such as dyslipidemia, glucose and high blood pressure are common in diabetic patients. Visit-to-visit variabilities in these measures have been reported as potential residual cardiovascular risk factors. However, the relationship between these variabilities and their effects on cardiovascular prognosis have not been studied.

**Methods:**

A total of 22,310 diabetic patients with ≥ 3 measurements of systolic blood pressure (SBP), blood glucose, total cholesterol (TC), and triglyceride (TG) levels during a minimum of three years at three tertiary general hospitals were selected. They were divided into high/low variability groups for each variable based on the coefficient of variation (CV) values. The primary outcome was the incidence of major adverse cardiovascular events (MACE), a composite of cardiovascular death, myocardial infarction, and stroke.

**Results:**

All high CV groups had a higher incidence of MACE than those with low CV (6.0% vs. 2.5% for SBP-CV groups, 5.5% vs. 3.0% for TC-CV groups, 4.7% vs. 3.8% for TG-CV groups, 5.8% vs. 2.7% for glucose-CV groups). In multivariable Cox regression analysis,, high SBP-CV (HR 1.79 [95% CI 1.54–2.07], p < 0.01), high TC-CV (HR 1.54 [95% CI 1.34–1.77], p < 0.01), high TG-CV (HR 1.15 [95% CI 1.01–1.31], p = 0.040) and high glucose-CV (HR 1.61 [95% CI 1.40–1.86], p < 0.01) were independent predictors of MACE.

**Conclusion:**

Variability of SBP, TC, TG and glucose are important residual risk factors for cardiovascular events in diabetic patients.

**Supplementary Information:**

The online version contains supplementary material available at 10.1186/s12933-023-01848-x.

## Background

Diabetes mellitus (DM) is often accompanied by other metabolic disorders such as dyslipidemia and hypertension; dyslipidemia affects up to 90% of type 2 diabetes patients and hypertension is found concomitantly in up to 70% of diabetic patients [1, 2]. Whilst diabetes mellitus (DM) itself represents a potent cardiovascular risk factor, the simultaneous presence of comorbidities frequently results in an increased susceptibility to adverse clinical outcomes amongst diabetic patients. The co-presence of hypertension and diabetes mellitus is associated with a mortality rate and incidence of cardiovascular events that are elevated by 44% and 41%, respectively, compared to 7% and 9% for those without hypertension [3]. Diabetic dyslipidemia, often characterised by hypertriglyceridemia and low high-density lipoprotein cholesterol (HDL-C), is associated with higher risks of adverse cardiovascular outcomes [4].

Individual parameters such as systolic blood pressure (SBP), lipid profile and glucose levels are often prone to visit-to-visit variability. Recent evidence suggests that variabilities of aforementioned parameters increase adverse cardiovascular outcomes including all-cause mortality, stroke and heart failure [5–8]. Extensive data on variabilities of metabolic parameters in diabetic population are, however, lacking.

Previously, the authors of the present study have shown that variability of triglyceride levels may be harmful in diabetic patients [9]. The presence of other metabolic profile variabilities in diabetic patients, and whether they independently exert influence on clinical outcomes is yet to be elucidated. Our study aims to assess the variability of SBP, total cholesterol (TC), triglyceride (TG) and blood glucose and their effects on cardiovascular outcomes.

## Methods

### Study design

This study was a multicenter retrospective cohort investigation using the Observational Medical Outcomes Partnership (OMOP) Common Data Model (CDM) database of three tertiary institutions in Korea (Korea University Anam Hospital, Korea University Guro Hospital and Korea Ansan Hospital). The Observational Health Data Sciences and Informatics cooperation offers the OMOP CDM schema, which is used to standardize hospital electronic health records in the OMOP CDM database [https://github.com/OHDSI/CommonDataModel]. In Korea, the ICD-10 coding system is used to classify diseases, and OMOP-CDM gives concept IDs uniquely matched to this code. Subsequently, the data were evaluated utilizing the OMOP-CDM concept ID, which was transferred to the ICD-10 code (Supplementary Table S1). The present study’s OMOP-CDM data were extracted via direct querying.

For the study population, 24,694 people who were 40 years old or older and whose SBP, serum TC, TG and glucose levels were measured at least three times, and the first measurement taken between January 2017 and June 2019 were chosen. The date of the first measurement was recorded as the index date for each patient. Variability was calculated based on these three-year values (the modeling phase). Patients who were not diabetic were excluded, as were those who had a known malignancy, myocardial infarction (MI), or stroke at baseline. Outcome events occurring only after the collection of at least three measurements of target parameters were recorded. Patients whose outcome event occurred before the measurement of target parameters (SBP, TC, TG and glucose) for three times or more, as specified above, were excluded. Patients with missing values, including serum creatinine, high density lipoprotein cholesterol (HDL-C), or urine albumin, were also excluded. Finally, a total of 22,310 patients were included. The Institutional Review Board of each participating center approved this study. Due to the retrospective study design and use of anonymized data, written informed permission was waived.

### Laboratory measurement and variability

Blood samples for routine laboratory tests were drawn during the day after an overnight fast. Using a uniform enzymatic colorimetric technique, serum lipid profile levels were determined. Blood pressure (BP) measurements were taken in an outpatient setting after 5 min of rest, and patients were advised not to drink caffeine or smoke cigarettes before BP measurements for at least 30 min. In order to determine the coefficient of variation (CV), we analyzed the measurements of SBP, TC, TG and glucose levels. The CV, as the variability index, was calculated as 100 × σ/µ, where σ is the standard deviation and µ is the mean of the measurements. The median CV for each measurement served as the dividing line between the high CV and low CV groups.

### Study variables and outcomes

Hypertension was defined as systolic blood pressure ≥ 140 mmHg or diastolic blood pressure ≥ 90 mmHg, anti-hypertensive drug use, or OMOP-CDM concept ID for hypertension. Diabetes mellitus was defined as a fasting plasma glucose level ≥ 126 mg/dL, a HbA1c level ≥ 6.5%, anti-diabetic drug use, or OMOP-CDM concept ID for diabetes mellitus. Dyslipidemia was defined as serum TC ≥ 240 mg/dL, LDL-C ≥ 160 mg/dL, TG ≥ 200 mg/dL, or HDL-C < 40 mg/dL, taking lipid lowering drugs, or OMOP-CDM concept ID for dyslipidemia. Based on the Modification of Diet in Renal Disease equation, chronic kidney disease was defined as a glomerular filtration rate of less than 60 mL/min/1.73 m^2^. Patients on antihypertensive drugs, statins, TG-lowering drugs such as fenofibrates and omega-3 fatty acids, antiplatelets, and anticoagulants were defined as those who were prescribed the medications with a proportion of days covered (PDC) of 50% during the modeling phase. Information on alcohol consumption and smoking status were obtained from patient-reported medical records. Smoking status was classified as current smoker or never/previous smoker. SCORE2 risk prediction algorithm was used to estimate cardiovascular risk for each patient, and the results were classified into low-moderate, high- and very high-risk groups [10].

The primary outcome was the occurrence of major adverse cardiovascular events (MACE), defined as a composite of cardiovascular death, new-onset MI and stroke. The dates and causes of death were extracted from death certificates in medical records. MI was determined by the presence of a serum creatinine kinase myocardial band (CK-MB) level over the upper limit of normal with a rising and/or falling pattern during hospitalization via the emergency department. Stroke was defined as having the matching OMOP-CDM concept ID or having a brain MRI that showed an acute, subacute, or recent cerebral infarction. The entire follow-up duration was used to analyze the time-to-event outcomes, and patients were censored at the time of death or the last available follow-up.

### Statistical analysis

Categorical variables are reported as numeric values (percentages) and continuous variables are reported as mean ± standard deviation. The study compared categorical variables by using either the χ2 test or Fisher’s exact test, and continuous variables by using either Student’s t-test or Mann-Whitney U-test, as appropriate. Pearson’s correlation coefficient was used to assess the correlation between each metabolic parameter’s CV and mean values. The cumulative incidences were calculated using Kaplan-Meier censoring estimates. Multivariable Cox proportional hazard regression analysis was used to compute the hazard ratios (HRs) and 95% confidence intervals (CI). To determine the independent predictors of MACE, Cox proportional hazards analysis was performed to identify significant variables, which were subsequently included in backward stepwise multivariable analysis. The final multivariable model included age, sex, serum creatinine, dyslipidemia, prior history of MI, prior history of stroke, anti-hypertensive drugs, statins and insulin. A p-value of < 0.05 was considered statistically significant. All analyses were performed using SAS (version 9.4; SAS Institute Inc., Cary, NC, USA) and R Statistical Software (version 4.1.2; R Foundation for Statistical Computing, Vienna, Austria).

## Results

### Baseline characteristics

The baseline characteristics of the high-CV and low-CV groups for each variable are shown in Table 1. The high CV group consisted of more alcohol drinkers and more smokers compared with the low CV group, regardless of the variable. The high CV group consisted of more men for TC, TG and glucose, while high SBP-CV group consisted of less men compared with the low SBP-CV group. Age showed varied proportions across the variables, as did hypertension, dyslipidemia, prior MI and prior stroke. The high CV group for each variable showed significantly higher CV values for all the other remaining variables, compared with the low CV group. The baseline characteristics for the total population are shown in **Supplemental Table S2**.

**Table 1:** Baseline characteristics of the study population according to low and high CV groups

	SBP-CV			TC-CV			TG-CV			Glucose-CV		
	Low (n = 11,155)	High (n = 11,155)	p-value	Low (n = 11,155)	High (n = 11,155)	p-value	Low (n = 11,155)	High (n = 11,155)	p-value	Low (n = 11,155)	High (n = 11,155)	p-value
Age (years)	62.7 ± 10.6	66.9 ± 10.9	< 0.01	65.5 ± 10.8	64.1 ± 11.1	< 0.01	66.6 ± 10.8	63.0 ± 10.9	< 0.01	64.7 ± 10.6	64.9 ± 11.4	0.10
Male (n, %)	6,512 (58.4)	5,852 (52.5)	< 0.01	6,066 (54.4)	6,298 (56.5)	< 0.01	5,771 (51.7)	6,593 (59.1)	< 0.01	6,005 (53.8)	6,359 (57.0)	< 0.01
Alcohol (n, %)	2,580 (23.1)	2,801 (25.1)	< 0.01	2,478 (22.2)	2,903 (26.0)	< 0.01	2,471 (22.2)	2,910 (26.1)	< 0.01	2,495 (22.4)	2,886 (25.9)	< 0.01
Smoking (n, %)	2,104 (18.9)	2,430 (21.8)	< 0.01	2,020 (18.1)	2,514 (22.5)	< 0.01	2,067 (18.5)	2,467 (22.1)	< 0.01	1,922 (17.2)	2,612 (23.4)	< 0.01
Hypertension (n, %)	8,219 (73.7)	9,500 (85.2)	< 0.01	8,901 (79.8)	8,818 (79.1)	0.17	9,030 (90.0)	8,689 (77.9)	< 0.01	8,638 (77.4)	9,081 (81.4)	< 0.01
Dyslipidemia (n, %)	10,025 (89.9)	10,160 (91.1)	< 0.01	10,069 (90.3)	10,116 (90.7)	0.28	10,059 (90.2)	10,126 (90.8)	0.13	10,069 (90.3)	10,116 (90.7)	0.28
Prior MI (n, %)	406 (3.6)	487 (4.4)	< 0.01	448 (4.0)	445 (4.0)	0.92	460 (4.1)	433 (3.9)	0.36	403 (3.6)	490 (4.4)	< 0.01
Prior stroke (n, %)	1,271 (11.4)	2,006 (18.0)	< 0.01	1,695 (15.2)	1,582 (14.2)	0.03	1,775 (15.9)	1,502 (13.5)	< 0.01	1,544 (13.8)	1,733 (15.5)	< 0.01
SCORE2 (n, %)			< 0.01			0.19			< 0.01			< 0.01
- low-moderate risk	5,128 (46.0)	3,356 (30.1)		4,267 (38.3)	4,217 (37.8)		3,840 (34.4)	4,644 (41.6)		4,604 (41.3)	3,880 (34.8)	
- high risk	3,841 (34.4)	3,944 (35.4)		3,928 (35.2)	3,857 (34.6)		3,881 (34.8)	3,904 (35.0)		3,921 (35.2)	3,864 (34.6)	
- very high risk	2,186 (19.6)	3,855 (34.6)		2,960 (26.5)	3,081 (27.6)		3,434 (30.8)	2,607 (23.4)		2,630 (23.6)	3,411 (30.6)	
SBP-CV (%)	6.5 ± 1.5	11.6 ± 2.7	< 0.01	8.6 ± 3.2	9.5 ± 3.5	< 0.01	9.0 ± 3.3	9.1 ± 3.4	< 0.01	8.4 ± 3.1	9.7 ± 3.5	< 0.01
TC-CV (%)	12.3 ± 7.1	14.0 ± 8.2	< 0.01	7.6 ± 2.3	18.7 ± 7.3	< 0.01	11.5 ± 6.6	14.7 ± 8.5	< 0.01	11.9 ± 7.0	14.4 ± 8.2	< 0.01
TG-CV (%)	28.7 ± 15.4	29.3 ± 15.7	< 0.01	25.7 ± 13.0	32.3 ± 17.1	< 0.01	18.1 ± 5.4	39.9 ± 14.8	< 0.01	27.4 ± 14.6	30.6 ± 16.3	< 0.01
Glucose-CV (%)	16.5 ± 11.5	21.9 ± 14.8	< 0.01	16.8 ± 12.0	21.6 ± 14.6	< 0.01	17.9 ± 12.8	20.5 ± 14.2	< 0.01	9.3 ± 3.5	29.1 ± 12.7	< 0.01
Baseline SBP (mmHg)	128.6 ± 12.4	130.6 ± 18.1	< 0.01	128.9 ± 14.9	130.4 ± 16.2	< 0.01	129.3 ± 15.4	129.9 ± 15.7	< 0.01	128.6 ± 14.7	130.6 ± 16.4	< 0.01
Baseline TC (mg/dL)	157.2 ± 36.6	156.6 ± 45.2	0.260	149.9 ± 30.4	164.0 ± 48.6	< 0.01	152.8 ± 36.1	161.0 ± 45.2	< 0.01	157.4 ± 35.7	156.4 ± 45.9	0.075
Baseline TG (mg/dL)	147.8 ± 98.6	150.1 ± 103.1	0.088	134.2 ± 79.1	163.6 ± 116.9	< 0.01	130.6 ± 68.2	167.2 ± 122.6	< 0.01	141.0 ± 86.3	156.8 ± 113.1	< 0.01
Basline glucose (mg/dL)	141.1 ± 51.1	146.8 ± 65.0	< 0.01	138.0 ± 46.9	149.9 ± 67.7	< 0.01	140.3 ± 53.3	147.5 ± 63.1	< 0.01	129.5 ± 33.3	158.4 ± 73.0	< 0.01
Mean SBP (mmHg)	127.9 ± 9.5	128.6 ± 11.1	< 0.01	127.9 ± 10.2	128.5 ± 10.5	< 0.01	128.2 ± 10.5	128.3 ± 10.2	0.25	127.5 ± 10.0	128.9 ± 10.7	< 0.01
Mean TC (mg/dL)	149.8 ± 27.3	147.6 ± 28.6	< 0.01	146.3 ± 26.3	151.1 ± 29.5	< 0.01	146.2 ± 27.0	151.2 ± 28.7	< 0.01	150.8 ± 27.3	146.6 ± 28.6	< 0.01
Mean TG (mg/dL)	139.9 ± 70.8	142.7 ± 75.1	< 0.01	129.4 ± 59.1	153.3 ± 83.0	< 0.01	125.0 ± 54.8	157.6 ± 84.4	< 0.01	135.0 ± 65.0	147.7 ± 79.7	< 0.01
Mean glucose (mg/dL)	134.8 ± 27.1	137.8 ± 33.1	< 0.01	133.4 ± 27.5	139.2 ± 32.5	< 0.01	134.0 ± 28.0	138.7 ± 32.1	< 0.01	126.2 ± 21.8	146.4 ± 33.9	< 0.01
RAS blocker (%)	6,492 (58.2)	8,032 (72.0)	< 0.01	7,349 (65.9)	7,175 (64.3)	0.01	7,485 (67.1)	7,039 (63.1)	< 0.01	6,925 (62.1)	7,599 (68.1)	< 0.01
DHP-CCB (%)	4,639 (41.6)	5,962 (53.5)	< 0.01	5,301 (47.5)	5,300 (47.5)	0.99	5,496 (49.3)	5,105 (45.8)	< 0.01	5,049 (45.3)	5,552 (49.8)	< 0.01
Beta-blocker (%)	2,860 (25.6)	4,243 (38.0)	< 0.01	3,496 (31.3)	3,607 (32.3)	0.11	3,670 (32.9)	3,433 (30.8)	< 0.01	3,338 (29.9)	3,765 (33.8)	< 0.01
Diuretics (%)	3,857 (34.6)	5,212 (46.7)	< 0.01	4,504 (40.4)	4,565 (40.9)	0.41	4,712 (42.2)	4,357 (39.1)	< 0.01	4,216 (37.8)	4,853 (43.5)	< 0.01
Statin (%)	8,842 (79.3)	8,955 (80.3)	0.06	9,110 (81.7)	8,687 (77.9)	< 0.01	9,066 (81.3)	8,731 (78.3)	< 0.01	8,960 (80.3)	8,837 (79.2)	0.04
Insulin (%)	3,492 (31.3)	4,761 (42.7)	< 0.01	3,953 (35.4)	4,300 (38.6)	< 0.01	4,221 (37.8)	4,032 (36.2)	< 0.01	3,065 (27.5)	5,188 (46.5)	< 0.01

Categorical variables in n (%) and continuous variables in mean ± standard deviation

CCB, calcium channel blocker; CV, coefficient of variability; DHP, dihydropyridine; MI, myocardial infarction; RAS, renin-angiotensin system; SBP, systolic blood pressure; TC, total cholesterol; TG, triglyceride

### Correlations between variability of metabolic parameters

**Fig. 1:**
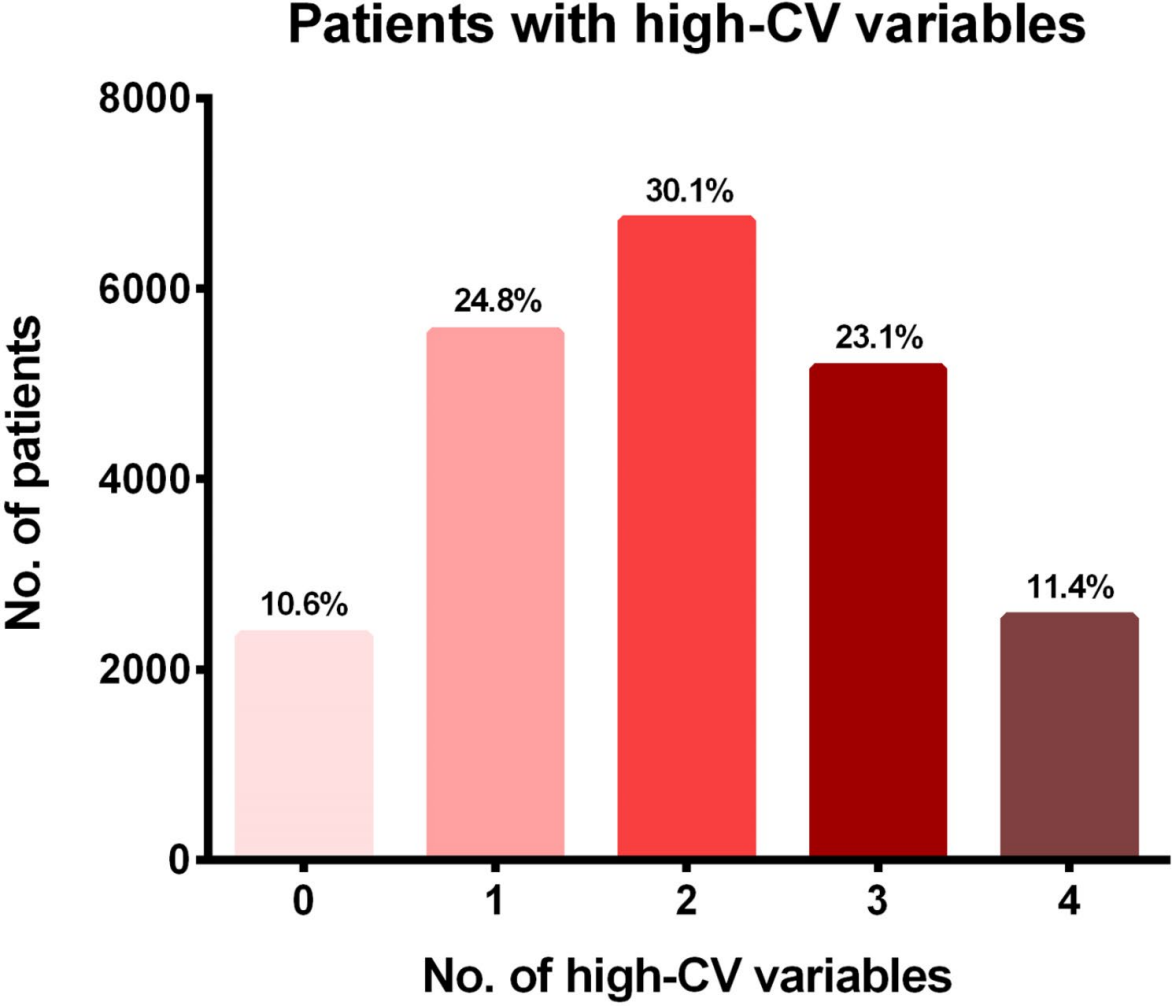
Percentage of patients with high-CV variables. CV, coefficient of variability

Over 3 years, the mean number of measurements was 15.1 ± 9.4 times for SBP, 7.8 ± 4.6 times for TC, 6.9 ± 3.9 times for triglyceride, and 8.6 ± 5.2 times for glucose (**Supplemental table S3**). Overall, 89.4% of the total patients had at least one high-CV parameter; 24.8% showed high variability in one parameter, and 30.1%, 23.1% and 11.4% in two, three and four parameters, respectiely (Fig. 1). Of the patients who had at least one high-CV variable, 13% exhibited high CV levels for all four variables, while 26% had high CV levels for three variables, and 35% had high CV levels for two variables. (Supplemental figure S1) Among patients with high SBP-CV, 85.6% also had at least one other high-CV variable, while 89.9% of high TC-CV patients, 86.3% of high TG-CV patients and 87.6% of high glucose-CV patients also showed high CV for at least one other variable (Supplemental figure S1).

The strength of correlation between each variable’s CV and other variables’ CV were investigated, as shown in **Supplemental Table S4A**. The between-variable correlation for CV was highest for TG and TC (r = 0.27), with the second highest being SBP and glucose (r = 0.25) and lowest for SBP and TG (r = 0.02). The between-variable correlation for mean values of each variable was highest for TC and TG (ρ = 0.31) and lowest for glucose and TC (ρ = 0.01) (**Supplemental table S4B**).

### Cumulative incidence of major adverse events

**Fig. 2:**
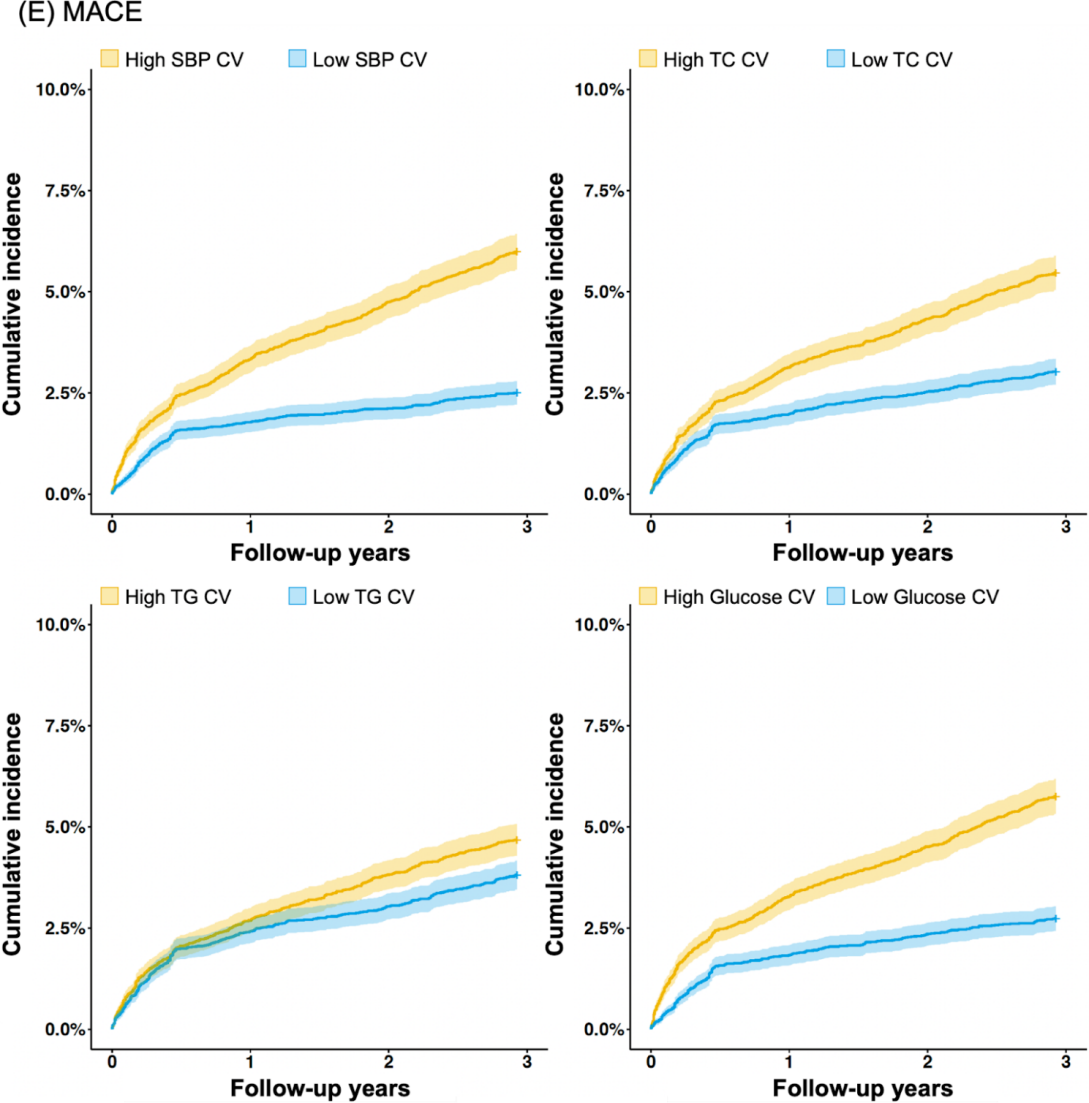
Major adverse cardiovascular events among patients with high and low CV. CV, coefficient of variability; HR, hazard ratio; SBP, systolic blood pressure; TC, total cholesterol; TG, triglycerides

The clinical outcomes occurring from +28 days post the index date were recorded, for a median duration of 1095 days. The cumulative incidences of the clinical outcomes are summarised in Table 2. The relationships between the occurrence of major adverse cardiovascular events and high and low CV of each metabolic variable are illustrated by Kaplan-Meier curves in Fig. 2. High CV was consistently associated with significantly higher MACE occurrence, with the highest difference of cumulative incidence between high and low SBP CV groups (6.0% vs. 2.5%, p < 0.01). High TC CV (5.5% vs. 3.0%, p < 0.01), high TG (4.7% vs. 3.0%, p < 0.01) and high glucose CV (5.8% vs. 2.7%) were also associated with higher risk of MACE occurrence. MI and stroke were also more prevalent in high-CV groups for all groups, while cardiovascular death (CVD) was more prevalent in high-CV groups for SBP-CV only. Out of SBP, TC, TG and glucose, SBP was the only variable for which high-CV group showed worse outcome compared to low-CV group in all of MACE, all-cause death, MI and stroke.

**Table 2:** Cumulative incidence of clinical outcomes

	SBP CV			TC CV			TG CV			Glucose CV		
	Low	High	p-value	Low	High	p-value	Low	High	p-value	Low	High	p-value
**MACE**	279 (2.5)	668 (6.0)	< 0.01	337 (3.0)	610 (5.5)	< 0.01	425 (3.8)	522 (4.7)	< 0.01	305 (2.7)	642 (5.8)	< 0.01
**MI**	239 (2.1)	549 (4.9)	< 0.01	286 (2.6)	502 (4.5)	< 0.01	351 (3.2)	437 (3.9)	< 0.01	252 (2.3)	536 (4.8)	< 0.01
**Stroke**	39 (0.4)	132 (1.2)	< 0.01	51 (0.5)	120 (1.1)	< 0.01	79 (0.7)	92 (0.8)	0.32	52 (0.5)	119 (1.1)	< 0.01
**CVD**	6 (0.05)	18 (0.2)	0.01	9 (0.1)	15 (0.1)	0.22	13 (0.1)	11 (0.1)	0.68	9 (0.1)	15 (0.1)	0.22

CV, coefficient of variation; CVD, cardiovascular death; MACE, major adverse cardiovascular event; MI, myocardial infarction; SBP, systolic blood pressure; TC, total cholesterol; TG, triglyceride

### Multivariable Cox regression analysis

In multivariable analysis by the Cox regression model, high SBP-CV (HR 1.79 [95% CI 1.54–2.07], p < 0.01), high TC-CV (HR 1.54 [95% CI 1.34–1.77], p < 0.01) high TG-CV (HR 1.15 [95% CI 1.01–1.31], p = 0.040) and high glucose-CV (HR 1.61 [95% CI 1.40–1.86], p < 0.01) were independent predictors of MACE (Table 3). High mean TC level was inversely related (HR 0.62 [95% CI 0.53–0.73], p < 0.01). The risk posed by mean SBP, mean TG and mean glucose levels did not reach statistical significance. Age (HR 1.01 [95% CI 1.01–1.02], p < 0.01), male sex (HR 1.35 [95% CI 1.17–1.54], p < 0.01), high serum creatinine (HR 1.10 [95% CI 1.07–1.14], p < 0.01), prior MI (HR 19.57 [95% CI 17.09–22.40], p < 0.01) and the use of anti-hypertensive drugs (HR 1.74 [95% CI 1.35–2.24], p < 0.01) were also associated with increased risk of MACE.

**Table 3:** The risk of MACE occurrence by multivariable analysis

	Univariable		Final multivariable model	
	HR (95% CI)	p-value	HR (95% CI)	p-value
**Age**	1.02 (1.01–1.03)	< 0.01	1.01 (1.01–1.02)	< 0.01
**Male sex**	1.74 (1.52-2.00)	< 0.01	1.35 (1.17–1.56)	< 0.01
**Smoking**	1.65 (1.01–1.03)	< 0.01		
**Creatinine**	1.17 (1.15–1.20)	< 0.01	1.10 (1.07–1.14)	< 0.01
**Hypertension**	2.67 (2.13–3.33)	< 0.01		
**Dyslipidemia**	1.96 (1.47–2.61)	< 0.01		
**Prior MI**	23.65 (20.76–26.93)	< 0.01	19.57 (17.09–22.40)	< 0.01
**Prior stroke**	1.50 (1.28–1.76)	< 0.01		
**Risk score (high risk)**	1.91 (1.60–2.27)	< 0.01		
**Risk score (very high risk)**	2.86 (2.41–3.39)	< 0.01		
**Baseline SBP**	1.01 (1.00-1.01)	0.024		
**Baseline TC**	1.00 (0.99-1.00)	0.875	1.45 (1.25–1.68)	< 0.01
**Baseline TG**	1.00 (1.00–1.00)	< 0.01		
**Baseline glucose**	1.00 (1.00–1.00)	< 0.01		
**High SBP-CV**	2.43 (2.11–2.79)	< 0.01	1.79 (1.54–2.07)	< 0.01
**High TC-CV**	1.83 (1.50–2.06)	< 0.01	1.54 (1.34–1.77)	< 0.01
**High TG-CV**	1.23 (0.85–1.97)	< 0.01	1.15 (1.01–1.31)	0.040
**High glucose-CV**	2.13 (1.55–2.27)	< 0.01	1.61 (1.40–1.86)	< 0.01
**High mean SBP**	0.99 (0.88–1.13)	0.935		
**High mean TC**	0.55 (0.48–0.63)	< 0.01	0.62 (0.53–0.73)	< 0.01
**High mean TG**	1.19 (1.05–1.35)	< 0.01		
**High mean glucose**	1.33 (1.17–1.52)	< 0.01		
**Anti-hypertensive drug**	3.79 (2.97–4.82)	< 0.01	1.74 (1.35–2.24)	< 0.01
**Statin**	1.72 (1.43–2.08)	< 0.01		
**Insulin**	1.60 (1.41–1.82)	< 0.01		

Multivariable analyses by Cox regression

CI, confidence interval; CV, coefficient of variability; HR, hazard ratio; MACE, major adverse cardiovascular events; MI, myocardial infarction; SBP, systolic blood pressure; TC, total cholesterol; TG, triglycerides

## Discussion

In this multicenter cohort study of 22,310 diabetic patients, we explored the relationships amongst four pertinent metabolic variables with regards to variability and the clinical outcomes thereof. Our main findings were that (1) high variability in one metabolic variable is associated with high variability in other metabolic variables; (2) variabiltiy appears to be more effective in predicting MACE compared to mean values; and (3) high variability groups tended to consist of more alcohol consumers and smokers compared with low CV groups.

Variability as a risk factor for adverse cardiovascular outcomes is increasingly being recognised. However, the interaction between variability of different metabolic variables on the effect of cardiovascular outcomes is not clear. A nationwide cohort study based on the National Health Insurance System in Korea compared the variabilities of blood pressure, glucose and cholesterol concentrations and body mass index, but while each variable was associated with the occurrence of adverse events, the correlation between the variables were not strong (maximum correlation coefficient r = 0.105, between TC and BMI variabilities) [11]. There is some evidence, however, that interactions between variability of different risk factors exist. A study found that there is an additive effect of high variability in both HbA1c levels and SBP on the risk of mortality in patients with type 1 diabetes [12]. The hazard ratio for high HbA1c variability was 1.78 and 1.69 for high SBP variability;, when high HbA1c and high SBP variability were both present, however, the hazard ratio was significantly higher at 2.37 [12]. Other studies have also reported additive effects of different metabolic variables on adverse outcomes such as mortality and renal diseases [13, 14]. Results from our study suggest that at least some interaction between different metabolic variables exist, as shown by the high percentage of patients who exhibit high variability for multiple variables compared with those who show variability in one variable (24.8% with one high-CV variable vs. 64.6% with two or more high-CV variables, Fig. 1). The absolute magnitude between the variables depicted by Pearson correlations were not strong, however, which suggests that there may be other metabolic variables or epidemiologic factors which interact to affect the variabilities. Nonetheless, it seems clear that variability of one parameter is often found concomittantly with another, which suggests they may exert synergistic effects upon each other.

The evaluation of risk of MACE occurrence in our study showed that high variability of SBP, TC, TG and glucose all had an advantage over the respective mean values in prediction of MACE occurrence. High SBP variability, in particular, exhibited a near twofold increae in MACE risk, while high mean SBP did not show significance in univariable analysis. Previous studies on have shown that visit-to-vist SBP variability is a strong predictor of adverse coronary events and stroke, independent of mean SBP levels [15–17]. A recent meta-analysis showed that SBP vatiability is associated with significantly increased risk of all-cause mortality and MACE in patients with type 2 diabetes [18]. SBP variability in diabetic patients is also asociated with increaesed mortality and other cardiovascular outcomes, and also macrovascular and microvascular complications [19, 20].

In our study, high TC variability was also associated with a 54% increase in MACE risk. TC variability was first recognised as a cardiovascular risk factor in the Framingham study [21]. High TC variability was an independent predictor of mortality and MACE in a nationwide cohort study based in South Korea [22]. Subgroup analyses from the study showed that high TC variability in diabetic subgroup was associated with increased stroke and MI but not all-cause mortality [22]. A recent population cohort study also reported that high total cholesterol variability was associated with a 20% increased CVD risk [23]. These results support our findings, and our data additively suggests that having a high TC variability may present with a higher risk of MACE than having a high mean TC value. Interestingly, a high mean TC level was associated with a reduced HR of 0.62. The inverse relationship between mean TC level and MACE in our study could be reflective of the fact that patients with high cardiovascular risks would likely be prescribed much stronger lipid-lowering agents to achieve the lower LDL-C target goals. This is in part explained by the fact that compared with mean TC level, baseline TC level is positively associated with MACE (HR 1.45 [95% CI 1.25–1.68], p < 0.01). The mean TC level in our study population was 141.3 mg/dL, which is lower than the reported values of mean TC in other studies; –175 mg/dL in the TNT trial, 192.3 mg/dL by Kim et al. and 188.1 by Wang et al, which suggests an iatrogenic influence by drugs [22, 24, 25].

Regarding TG, high TG variability was recently shown to be an independent risk predictor for adverse cardiovascular outcomes including all-cause death and MI, as compared with cumulative exposure to high levels of TG [9]. While Koh et al. evaluated TG as a single variable, this study consolidates the adverse effect of TG variability by analysing TG variability together with variabilities of other metabolic parameters. A retrospective cohort study in Hong Kong found that variabilities of LDL-C, TC-to-HDL ratio, and TG were all associated with all-cause and cardiovascular mortality, although LDL-C variability showed the strongest association, and TG variability the weakest [26]. Of note, a study by Wang et al. suggested that variabilities of HDL-C, LDL-C and TC, but not TG, were predictive of all-cause and cardiovascular mortality [25]. The discrepancy in results may be attributed to the usage of 10% increase in variabilities by Wang et al. rather than high versus low variabilities; the relationship of TG variability with clinical outcomes may thus have a nonlinear component. Further investigation may be needed for elucidation [25].

Previous analyses also showed that variations in fasting plasma glucose (FPG) was an independent predictor of cardiovascular death and mortality in general population and also in diabetic population [8, 27, 28]. The results from our study suggests that glucose variability is a significant predictor of adverse outcomes in diabetic patients, even after adjusting for clinical variables and variabilities of other metabolic variables. In a sensitivity analysis using HbA1c variability (data not shown), HbA1c-CV was also a significant predictor of mortality and other adverse outcomes; the results of HbA1c variability was not used due to a high proportion of the population lacking in multiple measurements (three or more) required for analysis in the study.

Notably, baseline demographics from our study show that smoking and alcohol drinking were positively associated with high CV across the all four variables in interest. This is in contrast to other variables such as age or sex, which showed varied proportions for low and high CV groups. Cigarette smoking is known to be associated with BP variability both during the day and between visits, likely contributing to the detrimental cardiovascular effects of smoking [29, 30]. The influence of alcohol on BP variability is less clear, with some evidence that heavy drinking induces circadian BP variability but no known relationship between alcohol consumption and visit-to-visit BP variability. The effects of TC-CV and TG-CV are likewise not fully explored as of yet. Overall, the evidence for interaction between lifestyle modification and metabolic parameter variabilities is lacking in detail, and thus entails a need for further research into the field. Although the exact relationship between smoking and alcohol consumption and metabolic variabilities is not fully clear, it is likely that smoking and drinking would act upstream to influence variabilities, considering the nature of the parameters.

Our study has several limitations. First, being a retrospective cohort study, precautions should be taken for the interpretation of the results as the possibility of selection bias cannot be ignored. The relationships found in our study between metabolic variabilities and clinical outcomes, for example, should be understood as associations rather than cause-and-effect. This warrants future trials based on randomisation of the patients in order to further consolidate our findings. Second, the measurements of the four variables of interest, namely SBP, TC, TG and glucose, were not all taken simultaneously. However, as shown in **Supplemental table S3**, the number of measurements and the interval betweeen measurements were similar for TC, TG and glucose. Considering the laborous task of coming into the hospital and providing a blood sample, it is likely that many of the TC, TG and glucose measurements would have been taken at the same time. The number of SBP measurements is naturally larger than the others due to its less-invasive quality. Third, adherence to treatment was not checked. However, the fact that multiple measurements over more than three years were needed for eligibility indirectly suggests at least moderate compliance of the enrolled patients.

In conclusion, in diabetic patients, metabolic variability parameters such as SBP-CV, TC-CV, TG-CV and glucose-CV are important risk factors for cardiovascular events, independent of the respective mean values. The exact mechanisms by which variability of different parameters affect each other are yet to be elucidated in future studies.

## Electronic supplementary material

Below is the link to the electronic supplementary material.

Supplementary Material 1

## Data Availability

Under Korean law, personal health information cannot be taken out of the country without the consent of the study subjects. This study is a retrospective study that exempts the consent of the study subjects, and it is impossible to export the study dataset abroad.

## References

[1] Jelinek HF, Osman WM, Khandoker AH, Khalaf K, Lee S, Almahmeed W, Alsafar HS (2017). Clinical profiles, comorbidities and complications of type 2 diabetes mellitus in patients from United Arab Emirates. BMJ Open Diabetes Res Care.

[2] Abdissa D, Kene K (2020). Prevalence and determinants of Hypertension among Diabetic Patients in Jimma University Medical Center, Southwest Ethiopia, 2019. Diabetes Metab Syndr Obes.

[3] Emdin CA, Rahimi K, Neal B, Callender T, Perkovic V, Patel A (2015). Blood pressure lowering in type 2 diabetes: a systematic review and meta-analysis. JAMA.

[4] Kaze AD, Santhanam P, Musani SK, Ahima R, Echouffo-Tcheugui JB (2021). Metabolic Dyslipidemia and Cardiovascular Outcomes in type 2 diabetes Mellitus: findings from the look AHEAD study. J Am Heart Assoc.

[5] Diaz KM, Tanner RM, Falzon L, Levitan EB, Reynolds K, Shimbo D, Muntner P (2014). Visit-to-visit variability of blood pressure and cardiovascular disease and all-cause mortality: a systematic review and meta-analysis. Hypertension.

[6] Bangalore S, Breazna A, DeMicco DA, Wun CC, Messerli FH, Committee TNTS, Investigators (2015). Visit-to-visit low-density lipoprotein cholesterol variability and risk of cardiovascular outcomes: insights from the TNT trial. J Am Coll Cardiol.

[7] Boey E, Gay GM, Poh KK, Yeo TC, Tan HC, Lee CH (2016). Visit-to-visit variability in LDL- and HDL-cholesterol is associated with adverse events after ST-segment elevation myocardial infarction: a 5-year follow-up study. Atherosclerosis.

[8] Xu D, Fang H, Xu W, Yan Y, Liu Y, Yao B (2016). Fasting plasma glucose variability and all-cause mortality among type 2 diabetes patients: a dynamic cohort study in Shanghai, China. Sci Rep.

[9] Koh SM, Chung SH, Yum YJ, Park SJ, Joo HJ, Kim YH, Kim EJ (2022). Comparison of the effects of triglyceride variability and exposure estimate on clinical prognosis in diabetic patients. Cardiovasc Diabetol.

[10] group Sw, collaboration ESCCr (2021). SCORE2 risk prediction algorithms: new models to estimate 10-year risk of cardiovascular disease in Europe. Eur Heart J.

[11] Kim MK, Han K, Park YM, Kwon HS, Kang G, Yoon KH, Lee SH (2018). Associations of variability in blood pressure, glucose and cholesterol concentrations, and body Mass Index with Mortality and Cardiovascular Outcomes in the General Population. Circulation.

[12] Wightman SS, Sainsbury CAR, Jones GC (2018). Visit-to-visit HbA1c variability and systolic blood pressure (SBP) variability are significantly and additively associated with mortality in individuals with type 1 diabetes: an observational study. Diabetes Obes Metab.

[13] Ceriello A, De Cosmo S, Rossi MC, Lucisano G, Genovese S, Pontremoli R, Fioretto P, Giorda C, Pacilli A, Viazzi F (2017). Variability in HbA1c, blood pressure, lipid parameters and serum uric acid, and risk of development of chronic kidney disease in type 2 diabetes. Diabetes Obes Metab.

[14] Hashemi Madani N, Ismail-Beigi F, Khamseh ME, Malek M, Ebrahimi Valojerdi A (2017). Predictive and explanatory factors of cardiovascular disease in people with adequately controlled type 2 diabetes. Eur J Prev Cardiol.

[15] Rothwell PM, Howard SC, Dolan E, O’Brien E, Dobson JE, Dahlof B, Sever PS, Poulter NR (2010). Prognostic significance of visit-to-visit variability, maximum systolic blood pressure, and episodic hypertension. Lancet.

[16] Vidal-Petiot E, Stebbins A, Chiswell K, Ardissino D, Aylward PE, Cannon CP, Ramos Corrales MA, Held C, Lopez-Sendon JL, Stewart RAH (2017). Visit-to-visit variability of blood pressure and cardiovascular outcomes in patients with stable coronary heart disease. Insights from the STABILITY trial. Eur Heart J.

[17] Bangalore S, Fayyad R, Messerli FH, Laskey R, DeMicco DA, Kastelein JJ, Waters DD (2017). Relation of variability of low-density lipoprotein cholesterol and blood pressure to events in patients with previous myocardial infarction from the IDEAL trial. Am J Cardiol.

[18] Chiriaco M, Pateras K, Virdis A, Charakida M, Kyriakopoulou D, Nannipieri M, Emdin M, Tsioufis K, Taddei S, Masi S (2019). Association between blood pressure variability, cardiovascular disease and mortality in type 2 diabetes: a systematic review and meta-analysis. Diabetes Obes Metab.

[19] Hata J, Arima H, Rothwell PM, Woodward M, Zoungas S, Anderson C, Patel A, Neal B, Glasziou P, Hamet P (2013). Effects of visit-to-visit variability in systolic blood pressure on macrovascular and microvascular complications in patients with type 2 diabetes mellitus: the ADVANCE trial. Circulation.

[20] Cardoso CRL, Leite NC, Salles GF (2020). Prognostic importance of visit-to-visit blood pressure variability for micro- and macrovascular outcomes in patients with type 2 diabetes: the Rio de Janeiro type 2 diabetes cohort study. Cardiovasc Diabetol.

[21] Kreger BE, Odell PM, D’Agostino RB, Wilson PF (1994). Long-term intraindividual cholesterol variability: natural course and adverse impact on morbidity and mortality–the Framingham Study. Am Heart J.

[22] Kim MK, Han K, Kim HS, Park YM, Kwon HS, Yoon KH, Lee SH (2017). Cholesterol variability and the risk of mortality, myocardial infarction, and stroke: a nationwide population-based study. Eur Heart J.

[23] Manemann SM, Bielinski SJ, Moser ED, St Sauver JL, Takahashi PY, Roger VL, Olson JE, Chamberlain AM, Remaley AT, Decker PA (2023). Variability in lipid levels and risk for Cardiovascular Disease: an Electronic Health Record-Based Population Cohort Study. J Am Heart Assoc.

[24] LaRosa JC, Grundy SM, Waters DD, Shear C, Barter P, Fruchart JC, Gotto AM, Greten H, Kastelein JJ, Shepherd J (2005). Intensive lipid lowering with atorvastatin in patients with stable coronary disease. N Engl J Med.

[25] Wang MC, Li CI, Liu CS, Lin CH, Yang SY, Li TC, Lin CC (2021). Effect of blood lipid variability on mortality in patients with type 2 diabetes: a large single-center cohort study. Cardiovasc Diabetol.

[26] Wan EYF, Yu EYT, Chin WY, Barrett JK, Mok AHY, Lau CST, Wang Y, Wong ICK, Chan EWY, Lam CLK (2020). Greater variability in lipid measurements associated with cardiovascular disease and mortality: a 10-year diabetes cohort study. Diabetes Obes Metab.

[27] Wang A, Liu X, Xu J, Han X, Su Z, Chen S, Zhang N, Wu S, Wang Y, Wang Y. Visit-to-Visit Variability of Fasting Plasma Glucose and the Risk of Cardiovascular Disease and All-Cause Mortality in the General Population. J Am Heart Assoc 2017, 6(12). 10.1161/JAHA.117.006757 PMC5779006 29187392

[28] Yu JH, Han K, Park S, Lee DY, Nam GE, Seo JA, Kim SG, Baik SH, Park YG, Kim SM (2019). Effects of long-term glycemic variability on incident cardiovascular disease and mortality in subjects without diabetes: a nationwide population-based study. Med (Baltim).

[29] Stewart MJ, Jyothinagaram S, McGinley IM, Padfield PL (1994). Cardiovascular effects of cigarette smoking: ambulatory blood pressure and BP variability. J Hum Hypertens.

[30] Ragueneau I, Michaud P, Demolis JL, Moryusef A, Jaillon P, Funck-Brentano C (1999). Effects of cigarette smoking on short-term variability of blood pressure in smoking and non smoking healthy volunteers. Fundam Clin Pharmacol.

