# Supplementary material for: Effects of metabolic parameters’ variability on cardiovascular outcomes in diabetic patients
Source: Cardiovasc Diabetol. 2023 May 15;22:114. doi: 10.1186/s12933-023-01848-x (PMC10186656; doi:10.1186/s12933-023-01848-x)

**Supplemental table S1. OMOP-CDM concept ID**

| **Variable** | **OMOP-CDM concept ID** |
| --- | --- |
| SBP | 4152194 |
| DBP | 4154790 |
| Hypertension | 320128, 4028741, 4289933, 314378, 319034, 443919, 201313, 439696, 439695, 439694, 195556, 317895, 443771, 4110948, 4028806, 45769159, 319826, 4108213 |
| Anti-hypertensive drug | 1332527, 21141332, 974702, 1332497, 1337103, 1351559, 1351583, 1351587, 1321637, 1332494,19122209, 1332499, 40235487, 40235491, 42960852, 42960868, 974473, 974474, 19112606, 40167202, 42969135,  42960725, 42960735, 19023454, 19023453, 19050220, 19022242, 19022241, 19073094, 42950866, 42950863, 42950869, 42969118, 42969112, 42969115, 42969109, 42969102, 42969106, 42950421, 42950354, 42950393, 1332525, 40171917, 40171863, 40171884, 1308851, 19096678, 19096677, 1308874, 42950300, 1592858, 40174776, 21030573, 21097731, 19058101, 43275607, 42960860, 21601761, 42960864, 42925749, 42925752, 42925744, 40171661, 1337070, 40163142, 21070867, 42961744, 42961740, 1332419, 19073093, 19078080, 19078101, 40184187, 40184217, 19106542, 19106543, 36883910, 40184184, 19081025, 19074672, 1340161, 19074673, 42959999, 42960002, 42969040, 42969049, 42969067, 42969022, 42969031, 42969058, 42707639, 42707641, 42959791, 42959787, 40165789, 40165762, 19076924, 1353818, 19011549, 42963311, 42960018, 42960013, 42955514, 42955431, 19127433, 19127434, 19080128, 40185304, 40185276, 42948648, 42948651, 42948654, 42932547, 42932541, 42932544, 42932538, 44507582, 19106593, 19106594, 42929938, 42929951, 42929931, 1309071, 42801011, 42801015, 42952826, 19019309, 1318860, 19112979, 19101807, 43291777, 42960005, 42960008, 1305450, 1328585, 1328689, 19133612, 19133613, 46287343, 1353780, 42960035, 42960027, 42960031, 40163753, 40163760, 40174811, 19101751, 19101750, 21058150, 1326020, 19022948, 19022949, 19017656, 19015804, 19011548, 19134566, 1318859, 19112981, 1319942, 1319943, 19020063, 19113063, 40167843, 40167849, 40167852, 19102491, 42969132, 42969141, 42969126, 42969138, 42969129, 42969123, 19127432, 19096740, 19102171, 40069686, 19096752, 40163271, 40163275, 21041304, 21056210, 42968999, 42968981, 42968990, 42969008, 19102170, 1334461, 1334492, 42972637, 42972640, 42972631, 42972634, 42969082, 42969085, 42969088, 42969091, 42969094, 42969097, 19121182, 1332495, 35604949, 35604953, 35604961, 40224166, 40224172, 40224175, 42930395, 42930392, 974642, 19028935, 40165261, 40165245, 19028936, 42959698, 19107180, 974447, 19101748, 1353820, 42969152, 42969149, 42969157, 42969154, 42938510, 42938516, 42938513, 40171905 |
| Dyslipidemia | 437530, 437827, 440360, 438720, 437521, 432867, 435516, 437530, 4047784 |
| Statin | 19019116, 40175390, 40175394, 40175400, 41048773, 1539411, 1526479, 1539469, 1539407, 44506638, 44506641, 19112569, 19077499, 1545997, 19123592, 1545996, 1332497, 42972637, 19122209, 1545959, 43527029, 42972640, 42972631, 42972634, 43527032, 1332494, 1332499, 2057677, 2057662, 2057670, 2057647, 2057655, 40165245, 40165261, 40165253, 42969173, 42969291, 42969232, 42969040, 42969067, 42969132, 42969022, 42969141, 42969058, 42968976, 42969049, 42969129, 42969031, 2063250, 42969118, 42969135, 42969126, 42969138, 42969102, 42969085, 42969082, 42969112, 42969109, 2063242, 42969008, 42969149, 42969154, 2063246, 42968999, 42969106, 2064553, 42968981, 2064586, 42969152, 2063264, 2063260, 42969157, 42969115, 2065490, 2064498, 42969094, 2064532, 2066023, 2066020, 2066024, 2065486, 42969088, 42969091, 2066021, 2063256, 2065482, 42968990, 2063252, 40165642, 40165646, 40165638, 42932538, 42932541, 42932547, 42932544, 2054845 |
| TG-lowering drugs | 19101756, 1551838, 19077244, 44506638, 44506641, 2054845,19022958, 19121242, 42968976, 35143166 |
| Insulin | 19058398, 1596972, 19135264, 19078603, 19078559, 46234239, 19078558, 19078552, 35602725, 42902356, 46221558, 43518492, 42902587, 42902742, 42902821, 42902945, 41348912, 41349148, 41348682, 43275300, 42921644, 42921712, 42921721, 43297029, 46233971, 46233974, 46234047, 46234050, 46234234, 46234237 |
| Antiplatelet agents | 19103854, 42483115, 42949815, 19021575, 1112896, 1113143, 1718409, 19075601, 42949815, 40241188, 46287538, 40163724, 1350311, 1350332, 42926308, 42926360, 42935771 |
| Anticoagulant agents | 40163524, 40163554, 40228154, 35606208, 43013030, 43013026, 40244444, 40244448, 40241333, 45892856, 45892860, 45892850 |
| Glucose | 3004501 |
| HbA1c | 3007263 |
| Creatinine | 3016723, 3051825 |
| CK-MB | 3005785 |
| Total cholesterol | 3027114 |
| LDL-cholesterol | 3028437 |
| HDL-cholesterol | 3007070 |
| TG | 3022192 |
| MI | 434376, 438438, 438170, 438447, 441579, 312327, 436706, 4051874, 4270024,4108677, 4108218, 4108217 |
| Stroke | 4108952, 4078446, 4077958, 4077959, 4077201, 4111708, 432923, 4120104, 4049659, 4176892, 4218781, 4319328, 4326561, 4110185, 4110186, 43530727, 42535426, 436430, 4110189, 4110190, 46270031, 4110192, 4108356, 762933, 4111714, 443454, 373503, 374055, 437306, 4108357, 4112020, 4338523,  381591, 4045737, 4045738, 4046360, 4108360, 4110194, 4110195, 4111710,  4111711, 4301259, 379778, 443605, 4046089, 4046090, 4047747, 35610098 |
| DM | 443732, 4099216, 443731, 443729, 37016354, 43530689, 37017432, 45769905, 45769906, 4193704, 4226121, 45757499, 43530656, 201530, 43530690, 45757277, 43530685, 36717156, 37016768, 4222415, 37016349, 443734, 4228443, 45757363, 37018728, 4063043, 4198296, 4129519, 4196141, 43531010, 4140466, 443412, 4224709, 4225055, 435216, 37017431, 36715571, 4295011, 45769904, 201254, 318712, 200687, 4222553, 377821, 42538169, 4224254, 45763583, 201531, 37016767, 45763584, 45757507, 37018566, 4227210, 37017429, 40484648, 45769876, 4151281, 4063042, 45769832, 4096042, 4221933, 4224879, 4096671, 4096670, 4224419, 4099652, 443733, 4096041,201826 |

CK-MB, creatine kinase myocardial band; DBP, diastolic blood pressure; DM, diabetes mellitus; HbA1c, haemoglobin A1c; HDL, high density lipoprotein; LDL, low density lipoprotein; MI, myocardial infarction; SBP, systolic blood pressure; TG, triglyceride.

**Supplemental table S2. Baseline characteristics of the total population**

|  | **Total population**  **(n=22,310)** |
| --- | --- |
| Age (years) | 64.8 ± 11.0 |
| Male (n, %) | 12.64 (55.4) |
| Alcohol (n, %) | 16,929 (75.9) |
| Smoking (n, %) | 17,776 (79.7) |
| Hypertension (n, %) | 17,719 (79.4) |
| Dyslipidemia (n, %) | 20,185 (90.5) |
| Prior myocardial infarction (n,%) | 893 (4.0) |
| Prior stroke (n,%) | 3,277 (14.7) |
| SCORE2 (n, %)  low-moderate risk  high risk  very high risk | 8,484 (38.0)  7,785 (34.9)  6,041 (27.1) |
| SBP-CV (%) | 9.0 ± 34 |
| TC-CV (%) | 13.1 ± 7.7 |
| Triglyceride-CV (%) | 29.0 ± 15.5 |
| Glucose-CV (%) | 19.2 ± 13.5 |
| Mean SBP (mmHg) | 128.2 ± 10.4 |
| Mean total cholesterol (mg/dL) | 148.7 ± 28.0 |
| Mean triglyceride (mg/dL) | 141.3 ± 73.0 |
| Mean glucose (mg/dL) | 136.3 ± 30.2 |
| RAS blocker (%) | 14,524 (65.1) |
| DHP-CCB (%) | 10,601 (47.5) |
| Beta-blocker (%) | 7,103 (31.8) |
| Diuretics (%) | 9,069 (40.7) |
| Statin (%) | 17,797 (79.8) |
| Insulin (%) | 8,253 (37.0) |

Categorical variables in n (%) and continuous variables in mean ± standard deviation.

CCB, calcium channel blocker; CV, coefficient of variability; DHP, dihydropyridine; RAS, renin-angiotensin system; SBP, systolic blood pressure; TC, total cholesterol.

**Supplemental table S3. Measurement numbers and intervals of the metabolic parameters**

|  | SBP | TC | TG | Glucose |
| --- | --- | --- | --- | --- |
| Number of measurements | 15.1 ± 9.4 | 7.8 ± 4.6 | 6.9 ± 3.9 | 8.6 ± 5.2 |
| Mean interval between measurements (days) | 89.8 ± 53.0 | 179.8 ± 109.2 | 198.5 ± 118.0 | 167.5 ± 110.0 |
| Median interval between measurements (days) | 85.7 ± 54.2 | 173.5 ± 114.2 | 192.6 ± 122.6 | 161.8 ± 113.6 |

CV = coefficient of variation; SBP = systolic blood pressure; TC = total cholesterol, TG = triglyceride

**Supplemental Table S4A. Inter-group correlation for CV of metabolic parameters**

| *r* | SBP CV | TC CV | Triglyceride CV | Glucose CV |
| --- | --- | --- | --- | --- |
| SBP CV | 1 | 0.14 | 0.02 | 0.25 |
| TC CV |  | 1 | 0.27 | 0.20 |
| Triglyceride CV |  |  | 1 | 0.13 |
| Glucose CV |  |  |  | 1 |

*r = Pearson correlation coefficient*

CV = coefficient of variation; SBP = systolic blood pressure; TC = total cholesterol; TG = triglyceride

**Supplemental table S4B. Inter-group correlation for mean values of metabolic parameters**

| *r* | Mean SBP | Mean TC | Mean triglyceride | Mean glucose |
| --- | --- | --- | --- | --- |
| Mean SBP | 1 | 0.06 | 0.09 | 0.06 |
| Mean TC |  | 1 | 0.31 | 0.01 |
| Mean triglyceride |  |  | 1 | 0.22 |
| Mean glucose |  |  |  | 1 |

*r = Pearson correlation coefficient*

SBP = systolic blood pressure; TC = total cholesterol; TG = triglyceride

**Supplemental figure S1. Inter-group variability correlations for high-variability patients**


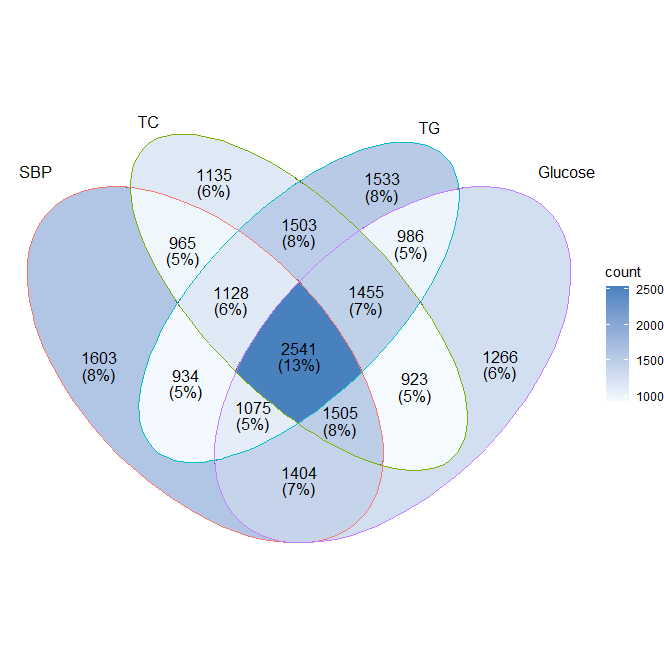


Patients with a high variability in at least one of the metabolic parameters were grouped according to their variability statuses.

SBP = systolic blood pressure; TC = total cholesterol; TG = triglyceride

**Supplemental figure S2. Kaplan-Meier curves**


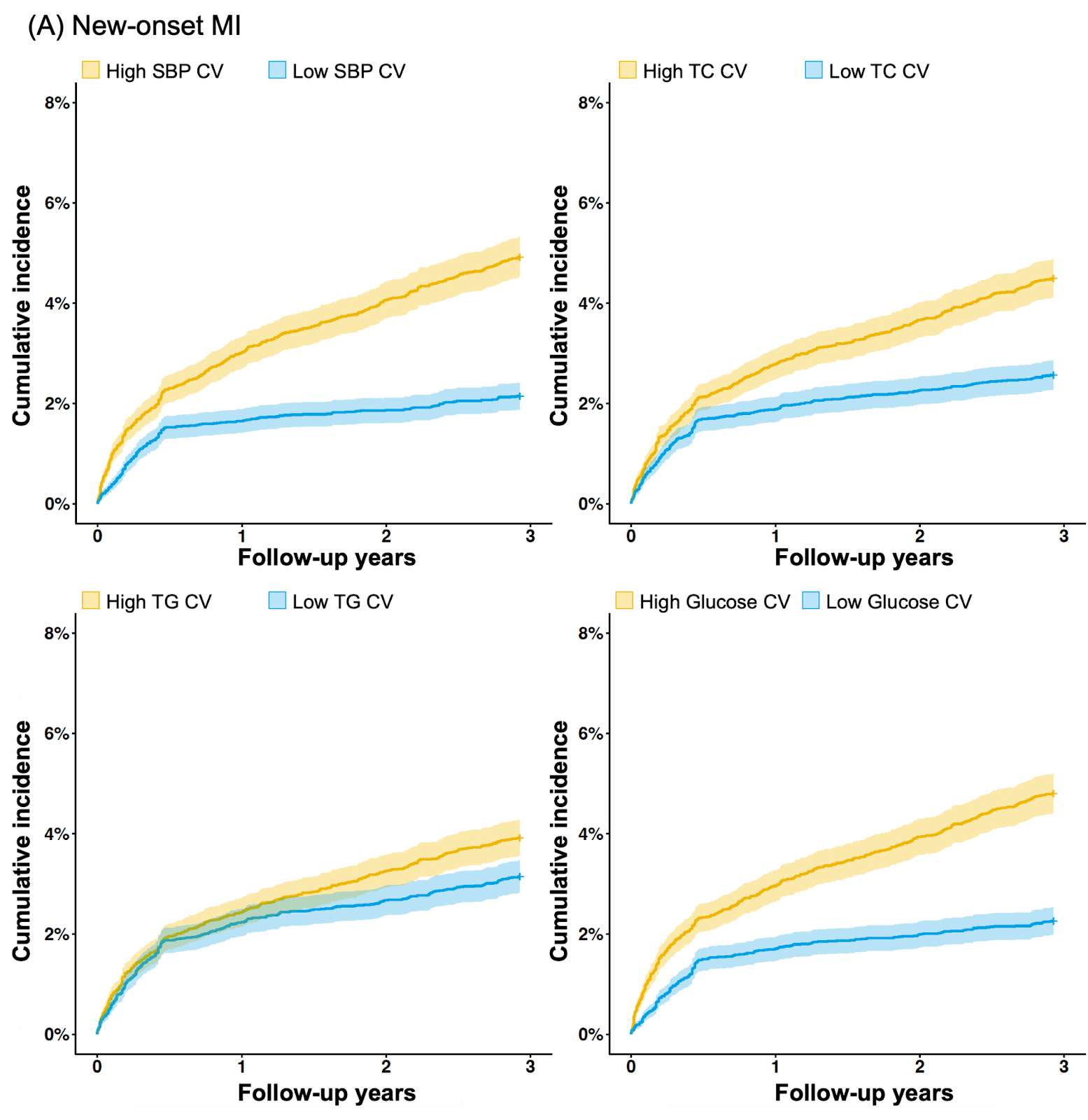


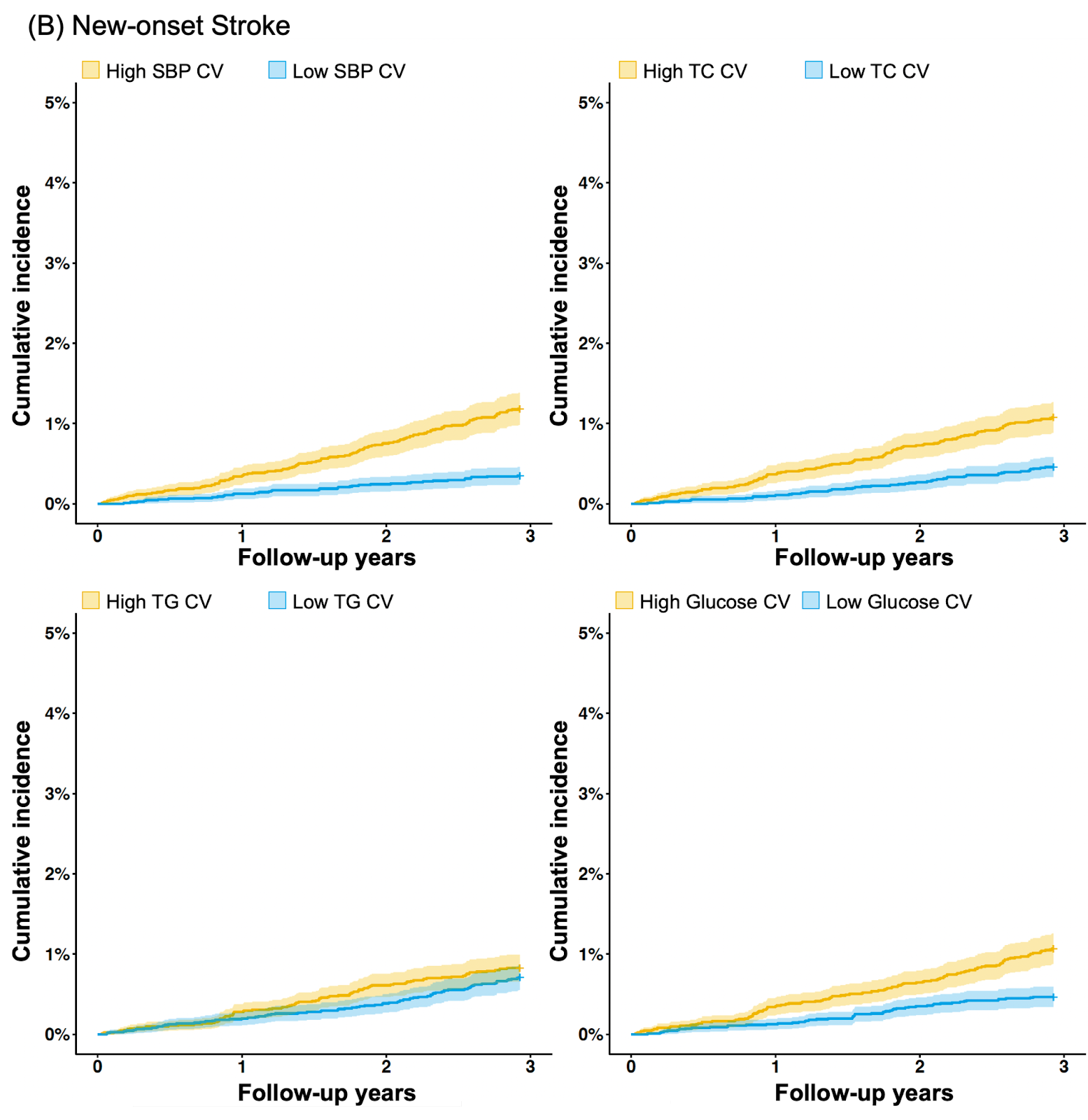


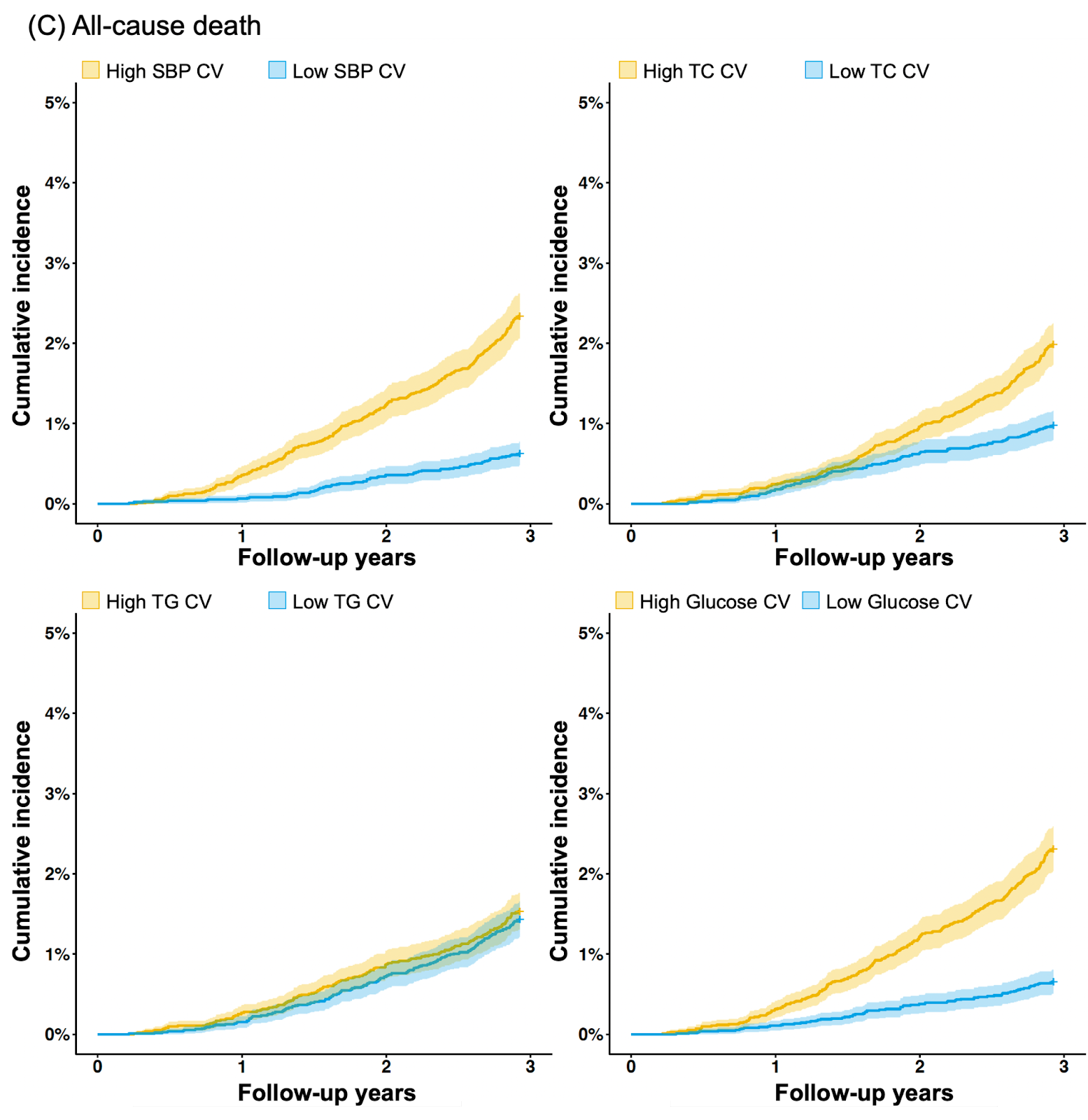


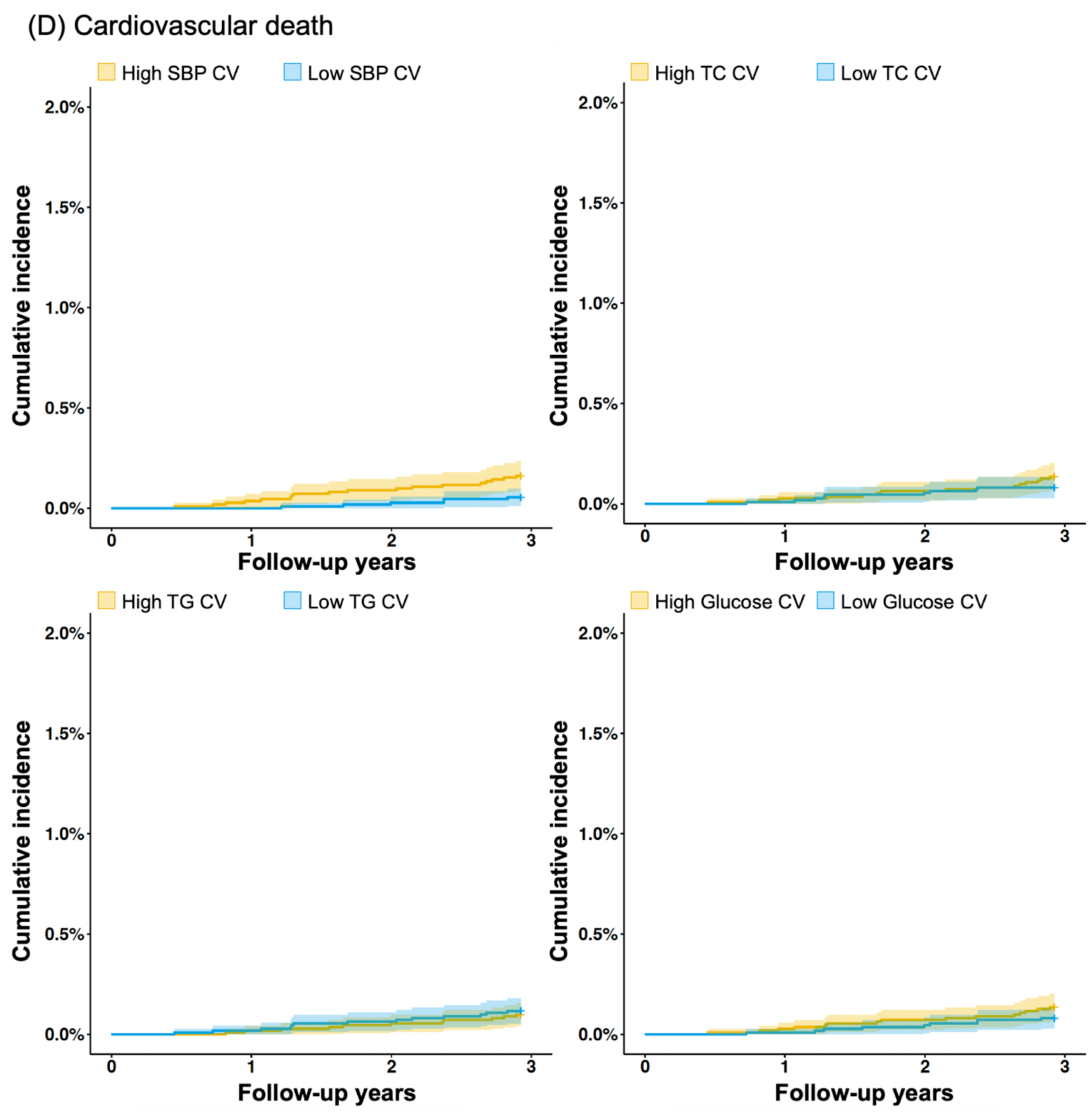

Supplement: Supplementary file 1 — Supplementary Material 1 [file 12933_2023_1848_MOESM1_ESM.docx]
